# Predicting Future Elective Colon Resection for Diverticulitis Using Patterns of Health Care Utilization

**DOI:** 10.5334/egems.193

**Published:** 2018-01-24

**Authors:** Lucas W. Thornblade, David R. Flum, Abraham D. Flaxman

**Affiliations:** 1 University of Washington, US

**Keywords:** Diverticulitis, General Surgery, Colorectal Surgery, Decision Making, Patient Preference

## Abstract

**Background:**

Recurrent diverticulitis is the most common reason for elective colon surgery and, although professional societies now recommend against early resection, its use continues to rise. Shared decision making decreases use of low-value surgery but identifying which patients are most likely to elect surgery has proven difficult. We hypothesized that Machine Learning algorithms using health care utilization (HCU) data can predict future clinical events including early resection for diverticulitis.

**Study Design:**

We developed models for predicting future surgery among patients with new diagnoses of diverticulitis (2009–2012) from the MarketScan® database. Claims data (diagnosis, procedural, and drug codes) were used to train three Machine Learning algorithms to predict surgery occurring between 52 and 104 weeks following diagnosis.

**Results:**

Of 82,231 patients with incident diverticulitis (age 51 ± 8 years, 52% female), 1.2% went on to elective colon resection. Using maximal training data (152 consecutive weeks of claims), the Gradient Boosting Machine model predicted elective surgery with an area under the curve (AUC) of 75% (95% uncertainty interval [UI] 71–79%). Models trained on less data resulted in less accurate prediction (AUC: 68% [64–74%] using 128 weeks, 57% [53–63%] using 104 weeks). The majority of resections (85%) were identified as low-value.

**Conclusion:**

By applying Machine Learning to HCU data from the time around a diagnosis of diverticulitis, we predicted elective surgery weeks to months in advance, with moderate accuracy. Identifying patients who are most likely to elect surgery for diverticulitis provides an opportunity for effective shared decision making initiatives aimed at reducing the use of costly low-value care.

## Introduction

Diverticular disease is common, affecting more than 50% of adults over age 60, of whom a third will go on to develop diverticulitis [1,2]. The burden due to diverticulitis in the United States is also significant, with over 300,000 admissions annually at a cost of more than $2.6 billion [3]. While diverticulitis can be treated with antibiotics, historically surgeons have recommended colectomy after only two episodes of diverticulitis [4]. However, guidelines from professional societies now strongly advise against treating patients with uncomplicated diverticulitis by elective colectomy [5]. This represents a shift in guidelines away from use of surgery in most cases. Despite these recommendations, rates of elective colon resection for uncomplicated diverticulitis have continued to increase [6]. Elective colon resection that is performed after fewer than three episodes of diverticulitis represents potentially low-value surgery and, to date, analyses have failed to identify driving factors for use of low-value surgery [7]. To our knowledge, prediction modeling has not previously been used to determine which patients are most likely to elect surgical resection for diverticulitis.

In the era of big data, there is growing interest in the automated use of patient data. Health care claims (e.g., diagnoses, procedures, drugs, and clinical variables) provide a wealth of diverse information pertaining to a patient’s clinical history [8,9,10]. However the heterogeneity and volume of this data have only limited utility with conventional analyses. For problems in health care that contain high-volume data with diverse variables, Machine Learning methods, which apply computer science algorithms to analyze complex non-linear relationships, can be applied towards prediction [11]. By exploiting variable diversity, sequence, and timing embedded in HCU data, Machine Learning algorithms may provide a more precise prediction than afforded by conventional regression-based models.

Patterns of prior health care utilization (HCU) may hold a key to predicting future health care events [11]. A patient’s unique pattern of existing diagnoses, past procedures and prescriptions, and frequency of outpatient visits and hospitalizations contains rich information about that patient’s state of health, symptom burden, and willingness to seek care. The hundreds of thousands of unique diagnostic, procedural and drug codes contained within health care claims provide a substrate that may be useful in prediction through advanced algorithms. Further, identifying high-volume users of the health care system may play a role in cost savings. To that end, HCU data remains an untapped resource. In prior work, we assessed whether hypothesis-driven variables (patient age, use of laparoscopy, or timing of disease recurrence) could explain the rise in elective surgery for diverticulitis but to-date, no compelling associations have been found [7]. In this study, we took an alternative approach and hypothesized that a patient’s unique pattern of HCU around the time of diagnosis of diverticulitis could be used to predict future elective surgery. We aimed to develop a model for predicting low-value care in diverticulitis by testing several Machine Learning algorithms. We also aimed to ascertain whether prediction modeling can be performed accurately as far as weeks to months in advance of an event. Future applications of robust prediction include informing patients for the purpose of shared decision making to reduce low-value surgery.

## Methods

Because prediction is typically most accurate when performed on large datasets, we selected commercial claims as a source of diverse data from a large cohort of patients. We tested prediction models on a retrospective cohort of adult patients (ages 18–64) with a new diagnosis of diverticulitis between 2009 and 2012 using the Thompson Reuters MarketScan® Commercial Claims database. Because of the de-identified nature of this data, it was not considered human subjects research and therefore this study was exempt from review by an Internal Review Board. MarketScan® includes person-level health care utilization data including inpatient, outpatient and pharmaceutical claims for employees and their dependents who are covered by employer-sponsored private health insurance and contains data from more than 30 million Americans annually. In this database, diagnoses are coded using the International Classification of Diseases, 9th Revision (ICD-9), drugs are coded using the National Drug Codes (NDC), and procedures are coded using the Current Procedural Terminology (CPT) system.

### Cohort Selection

We identified patients with a new diagnosis of diverticulitis using diagnosis codes (ICD-9: 562.11 & 562.13) between 2009 and 2012 who had been free from this disease during at least two years of continuous prior insurance enrollment (claims reviewed from 2007, onwards). In order to ensure equal follow-up, we included only patients with at least two years of continuous enrollment following diagnosis (claims followed through the end of 2014). Patients were excluded if they had any prior colon surgery or a diagnosis of colorectal cancer. Details of cohort selection are summarized in Appendix A.

### Defining HCU Time Periods

**Figure 1:**
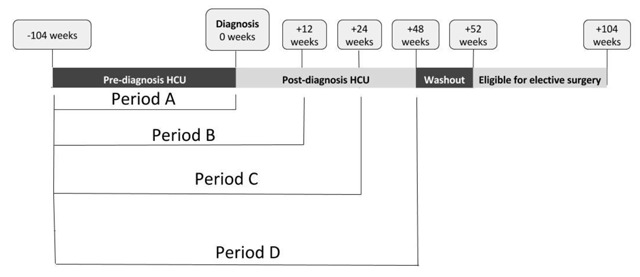
Timeline of health care utilization data in relation to diagnosis of diverticulitis and corresponding periods A–D.

In order to train models using HCU from both before and after a diagnosis of diverticulitis, we excluded any patients who had colon resection during the first 52 weeks after diagnosis. HCU data were sampled for any claim for elective colon resection between 52 and 104 weeks following the diagnosis of diverticulitis (Figure 1). To avoid including surgeries that are were potentially emergent (i.e. non-elective), patients were excluded if colon resections occurred within one week after an emergency room visit. We developed models using all inpatient, outpatient and pharmaceutical claims from four periods of varying length (A–D) around the time of diagnosis with diverticulitis: Period A) 104 weeks prior to diagnosis; Period B) 116 weeks (104 weeks pre-diagnosis to 12 weeks post-diagnosis; Period C) 128 weeks (104 weeks pre-diagnosis to 24 weeks post-diagnosis; and Period D) 152 weeks (104 weeks pre-diagnosis to 48 weeks post-diagnosis) (Figure 1). We specifically did not include HCU data from the 4 weeks before the period of eligibility for elective surgery (washout from 48 to 52 weeks post-diagnosis) because this period may include variables that are specific indicators of an upcoming surgery (e.g., prescription for bowel preparation, or pre-anesthesia and surgery clinic appointments etc.).

### Model Selection

To perform prediction using a large number of variables from data that are as diverse as health care claims, we selected a number of candidate Machine Learning models for testing in this study. Prior work by our group has identified these algorithms as candidates for prediction in claims data [12]. Random Forest (RF) is an algorithm made up of a number of regression trees that are created using bootstrap samples from training data [13], and predictions are made as aggregates of the estimates from all regression trees. Penalized logistic regression (PLR) is an algorithm based upon standard logistic regression but which applies a penalty to the absolute value of coefficients using the least absolute shrinkage and selection operator (LASSO) ultimately selecting certain variables for inclusion and thereby enhancing prediction of the final model [14,15,16]. Although penalized logistic regression is not strictly considered Machine Learning, because of this model’s capacity for handling many types of variables in prediction, it was included as a candidate model. Finally, Gradient Boosting Machine (GBM) is an algorithm that performs prediction via stage-wise assembly of weaker prediction models to develop a more precise estimate [18,19]. Links to source code for algorithms used in this study are available in Appendix B. Details and code for data processing and model selection for the purposes of reproduction are outlined in Appendix C.

### Predicting elective surgery

Using all available HCU data contained within MarketScan® claims as predictors, including codes for diagnoses, procedures, and drug prescriptions (including those not pertaining to diverticulitis), we compared the performance of three Machine Learning methods (RF, PLR, and GBM) [13,14,17] in their ability to predict which patients would go on to have elective colon surgery between 52 and 104 weeks following diagnosis. Unlike conventional modeling, no codes were pre-screened for use in each model. Instead, we exploited the capacity of Machine Learning algorithms for high-dimensional data by using all codes provided by claims. The ICD-9 and CPT procedural codes for colon surgery sampled from claims during this period are listed in Appendix A. While Machine Learning methods typically allow for some bias in order to minimize variance, all models were tuned for the purposes of maximizing predictive performance. In order to balance the tradeoff of bias and variance, model performance was measured by C-statistics based upon area under the receiver operating curves (AUC). Because of the very high volume of predictor variables applied in this study, we did not seek to define features of individual predictors.

Although the aim of this study was to determine the relative performance of several Machine Learning models, in order to determine levels of uncertainty, we applied a Train-Test-Validate approach [20]. We undertook internal validation to avoid diminishing sample size through subdivision of the cohort. This form of validation does not meet the standard of external validation as all training data were also used in validation. Models were validated using 10 replicates of 10-fold cross validation, where the cohort was randomly divided into 10 “folds”, the model was trained on 9 of them, and the performance was measured on the tenth [20]. Validation estimates were used to generate 95% Uncertainty Intervals (UI) (a correlate of the confidence interval) [21,22,23]. Missing data were treated as case-complete as these models were envisioned for application to existing HCU data.

### Identifying Low-Value Surgery

Based upon existing guidelines for colon resection in diverticulitis, we defined low-value surgery as an elective operation that occurred after two or fewer episodes of diverticulitis (either inpatient or outpatient) [5,7]. In order to identify surgeries that qualified as potentially low-value, we counted the number of episodes of diverticulitis preceding any colon surgery. Inpatient episodes are identified as a single inpatient claim for diverticulitis. Outpatient episodes were identified by an outpatient claim for diverticulitis and a prescription of ciprofloxacin and metronidazole within 1 week of the encounter. Antibiotic drug codes were identified from the MarketScan® RED BOOK™ Supplement. All subsequent diagnosis codes for diverticulitis that occurred within six-weeks of a prior episode were considered part of a single episode.

### Identifying Utilization Parameters

Using the highest performing prediction model, the cohort was divided into quintiles based upon predicted probability of undergoing future elective surgery. HCU data from a period of 2.5 years (104 weeks pre-diagnosis and 24 weeks post-diagnosis, Period C) was sampled for differences in utilization including the number of days in health care, number of emergency room visits, number of outpatient visits, number of unique diagnoses, number of prescriptions, number of claims for diverticulitis, and total health care costs. The differences in utilization between patients in the highest and lowest quintiles for likelihood of future surgery was assessed using t-tests (normally distributed continuous values), chi-squared tests (categorical values), and Mann-Whitney U tests (non-normal continuous values). All analyses were performed using free/libre open-source software (Python) [24,25].

## Results

**Figure 2:**
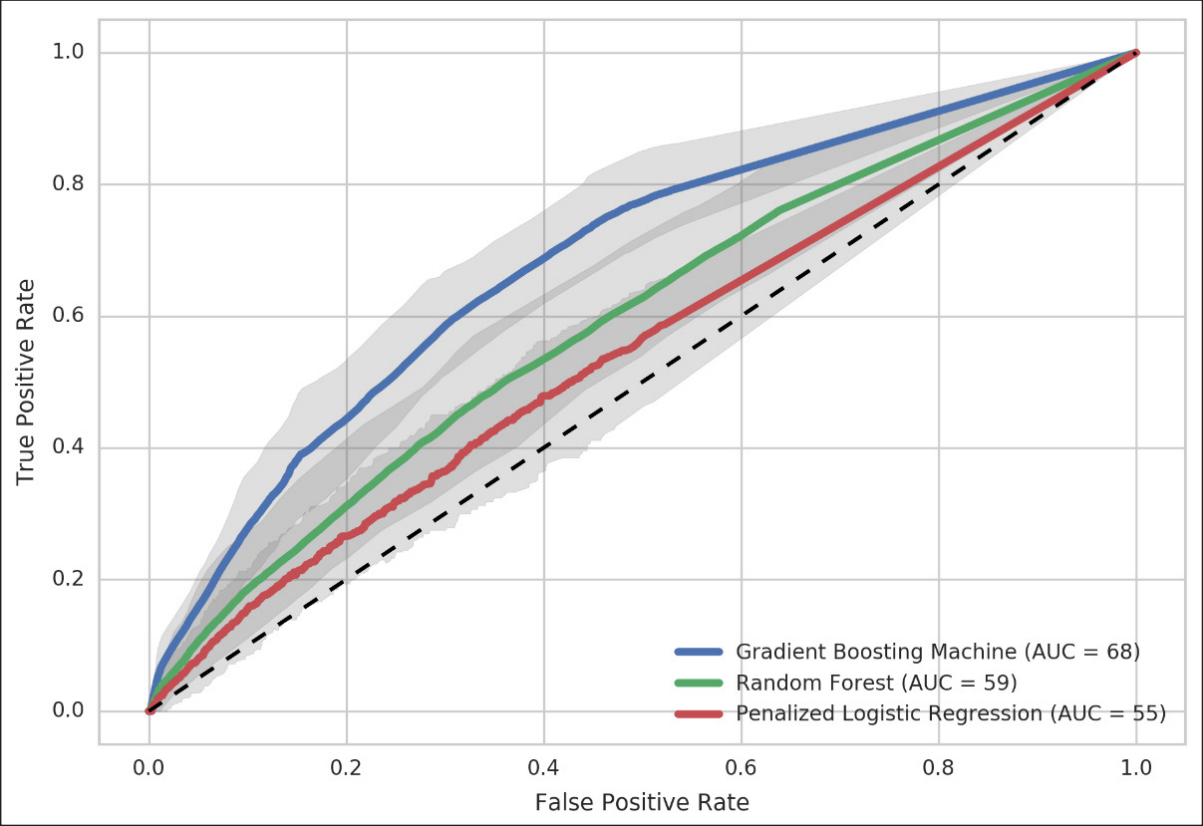
Receiver operating curves (ROC) of three machine learning algorithms using data from 104 weeks before to 24 weeks following diagnosis of diverticulitis: Gradient Boosting Machine, Random Forest, and Penalized Logistic Regression. Gray shading indicates 95% Uncertainty Interval.

We identified 82,231 adult patients (mean age 51 ± 8 years, 52% female) with incident diverticulitis between 2009 and 2012. Patient characteristics are summarized in Table 1. During the period of 52–104 weeks following diagnosis, 1.2% of patients went on to elective colon resection. Using 128 weeks of training data, we identified that the GBM method achieved the highest performance for predicting future surgery with an AUC of 68% (Uncertainty Interval (UI) 64–74%) compared with RF (59% [UI 53–65%]), and PLR (55% [UI 48–59%]) (Figure 2).

**Table 1:** Demographics and utilization parameters of patients in the lowest and highest quintiles for likelihood of undergoing future elective surgery for diverticulitis from the Gradient Boosting Machine algorithm.

	All patients (n = 84,791)	Lowest Likelihood Quintile (n = 16,958)	Highest Likelihood Quintile (n = 16,958)	p-value

**Demographics**				

age, mean ± SD	51.0 ± 8.6	51.3 ± 8.8	50.0 ± 8.7	**<0.001**
female, %	52.4%	54.9%	53.5%	0.18
Elective surgery, n (%)	947 (1.2%)	5 (0.3%)	49 (3.0)	
Utilization parameters*

Days in health care, mean ± SD	57.2 ± 46.9	57.0 ± 45.8	58.2 ± 46.0	0.34
ER visits, mean ± SD	1.1 ± 2.1	0.8 ± 2.0	1.8 ± 2.3	**<0.001**
Outpatient visits, mean ± SD	33.9 ± 30.4	34.3 ± 29.6	34.6 ± 31.1	0.96
Unique diagnoses, mean ± SD	29.8 ± 18.9	29.3 ± 18.7	30.8 ± 18.2	**<0.001**
Prescriptions, mean ± SD	43.4 ± 51.8	42.2 ± 50.3	43.4 ± 50.2	0.25
Diverticulitis claims, mean ± SD	7.6 ± 11.0	1.4 ± 1.4	22.9 ± 14.2	**<0.001**
Total health care costs, median (Interquartile Range)	–	$25,298 ($11,761–52,846)	$44,475 ($24,356–82,379)	**<0.001**

*For all health care data from 104 weeks prior to diagnosis until 24 weeks following diagnosis of diverticulitis.

**Figure 3:**
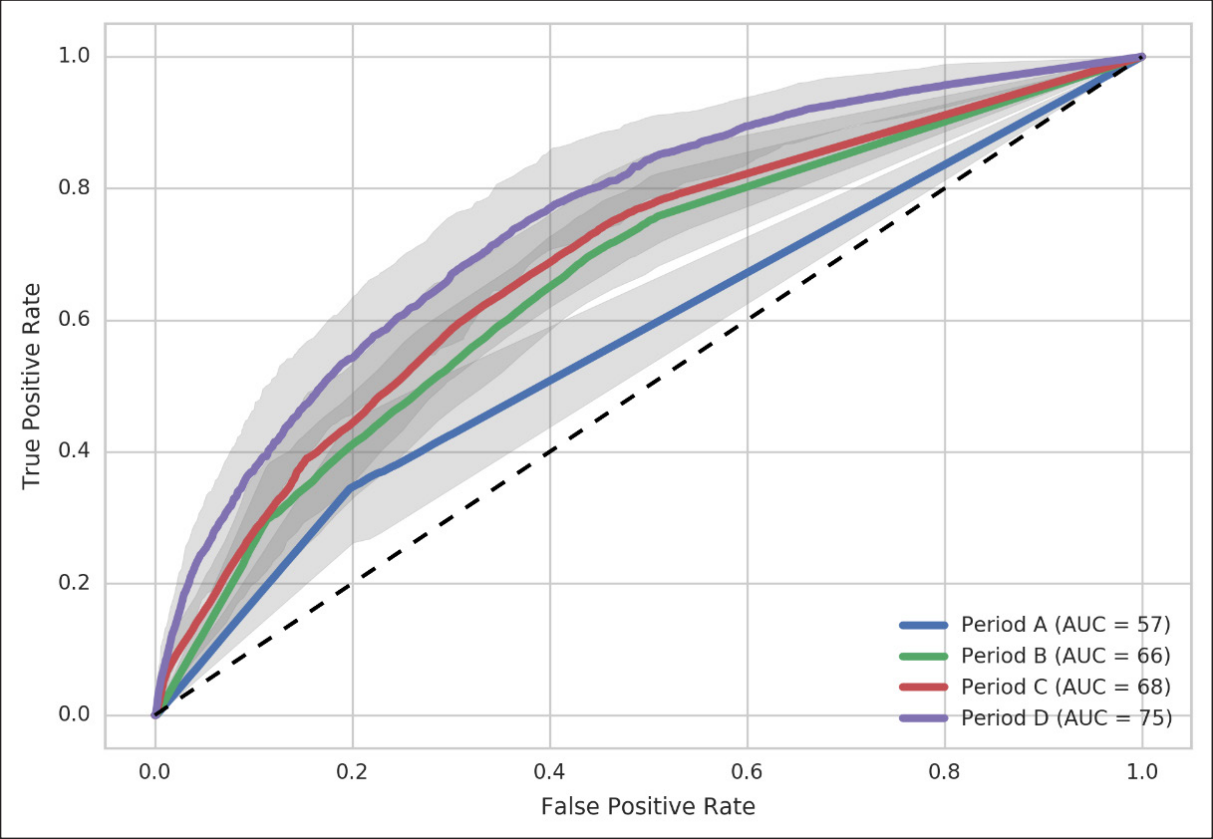
Receiver operating curves for Gradient Boosting Machine performance using data from Period A (104 weeks) Period B (116 weeks) Period C (128 weeks), and Period D (152 weeks). Gray shading indicates 95% Uncertainty Interval.

**Figure 4:**
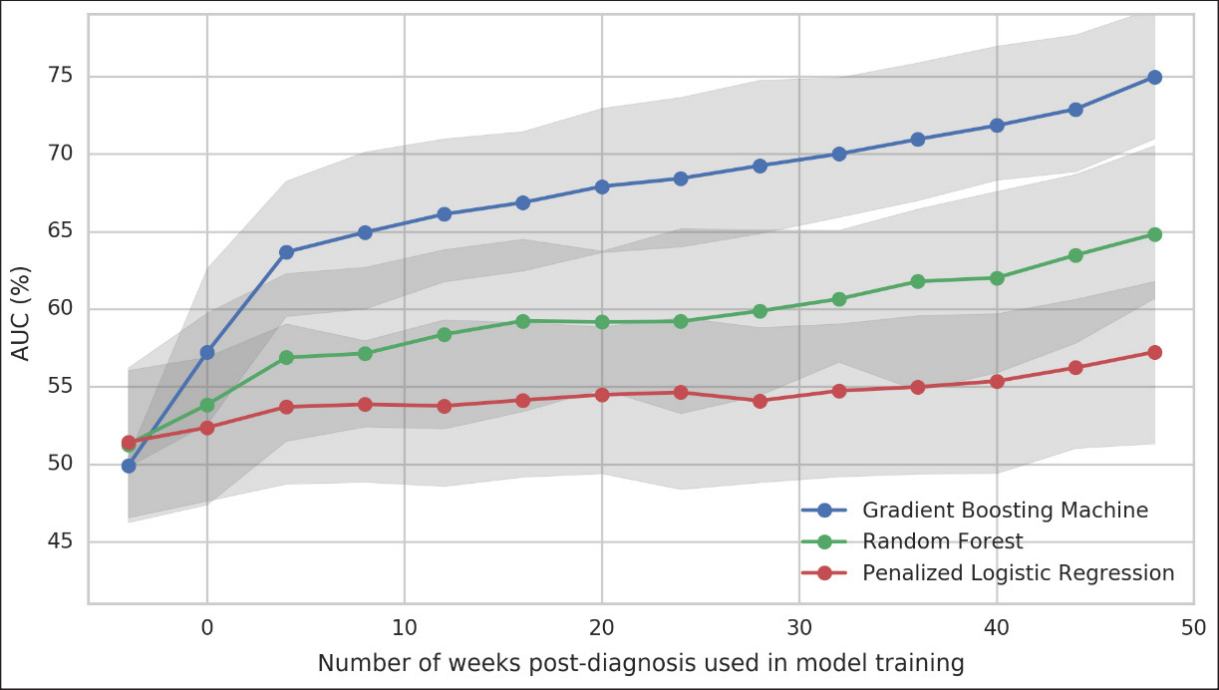
Trends of model performance with the addition of HCU data after diagnosis for four machine learning algorithms including Gradient Boosting Machine, Random Forest, and Penalized Logistic Regression. Gray shading indicates 95% Uncertainty Interval.

Using pre-diagnosis HCU data (Period A), the GBM model predicted elective surgeries with an AUC of 57% (UI 53–63%). Each addition of post-diagnosis HCU data led to greater prediction. GBM models trained on 116 weeks (Period B), 128 weeks (Period B) and 152 weeks (Period D) successfully predicted surgery with AUC of 66% (UI 62–71%), 68% (UI 64–74%) 75% (UI 71–79%) respectively (Figure 3). Figure 4 demonstrates continuous change in AUC with additional post-diagnosis HCU data. The most influential codes from each model are listed in Appendix D. Review of these codes did not reveal clear patterns either within or between models.

Of the 947 patients who went on to elective surgery between 52 and 104 weeks after diagnosis, 809 (85%) were identified as having low-value surgery with only two or fewer episodes of diverticulitis prior to resection (397 with two prior episodes, and 412 with only a single prior episode of diverticulitis).

Compared with the lowest quintile, patients in the highest quintile for likelihood of future elective surgery demonstrated more utilization of health care resources by several parameters (Table 1). High-likelihood patients had more emergency room visits (mean 1.8 vs. 0.8, p < 0.001), more unique diagnoses (mean 30.8 vs. 29.3, p < 0.001), more individual claims for diverticulitis (mean 22.9 vs. 1.4, p < 0.001), and greater median health care costs ($44,475 vs. $25,298, p < 0.001) during 128 continuous weeks of health care.

## Discussion

Through the application of Machine Learning methods to the diverse data present in health care claims around the time of a new diagnosis of diverticulitis, we can predict with moderate accuracy, future elective colon resection. In this study we found that, for all algorithms tested, longer periods of claims capture yielded better prediction model performance. Of the three types of Machine Learning methods examined, the GBM algorithm demonstrated the best performance possibly by capitalizing on the co-occurrences of codes and code types. We performed experiments testing the inclusion of sequence and timing embedded within claims data by encoding bigrams and trigrams of codes and found that they did not increase the model’s predictive power. The GBM model’s superior performance over other methods (e.g., RF and PLR) may be explained by its ability to search out the most informative combinations of codes. Importantly, the GBM method achieved prediction with AUC of 69% at 24 weeks after diagnosis of surgeries that occurred between 52 and 104 weeks after diagnosis. In other words, as far as six months in advance, HCU data can be used to predict patient behavior related to low-value care.

By predicting future health care events, we have not made inference about the factors associated with low-value surgery [11,26]. The Machine Learning algorithms used in this analysis can provide prediction based upon a wide variety of data features, and as such may not be sufficiently logical to be understood in an inferential framework. In order to further explain the drivers of low-value surgery, we examined utilization features of patients classified as either high- or low-likelihood for future surgery as determined by the prediction algorithm. When comparing these groups, those patients who are most likely to undergo an elective resection for diverticulitis appear to be high utilizers of health care. These patients have more frequent ER visits, carry a greater number of diagnoses, and have more health care claims related to diverticulitis. It may be these features of higher utilization which drive the prediction model in identifying future surgery. Concurrent to this work, our group is enrolling patients in a large prospective study of diverticulitis. By surveying both patients and surgeons about symptoms and preferences that influence decision-making around surgery, we aim to gain clinical and patient-level data that cannot otherwise be ascertained from claims data alone.

This novel application of Machine Learning algorithms to HCU data likely has implications in other realms of health care. However at present, predictive modeling in health care has limited applications. Prediction can refine disease prognosis, can be used for risk stratification, or can aid providers in clinical decision making. For diverticulitis, this type of prediction can also be used to inform decisions made by surgeons and patients. Diverticulitis is typically not life limiting and is considered a preference sensitive condition, meaning patients may make therapeutic choices based upon cost, symptom burden, lifestyle, or some other factors other than survival. For preference sensitive conditions with documented excess of HCU, predictive modeling may be applied in shared decision making. Aids for shared decision making have been shown to reduce rates of costly elective surgery in other common conditions [27,28]. The application of Machine Learning to existing health care utilization data presents a unique opportunity to inform decision making for patients and physicians choosing between surgery and continued non-operative management for uncomplicated diverticulitis. Importantly, the methods described here predicted health care events as much as six months in advance, providing ample time for implementation of a shared decision making tool. Early future application of this form of prediction includes an ongoing prospective mixed-methods study at our institution of patients with diverticulitis. This research aims to understand the clinical and personal reasons that physicians and patients choose surgery to treat this disease. To do so, however, would require real-time access to claims within an integrated health system.

The application of Machine Learning to health care claims is a novel approach to this type of data and does have several important limitations. First, claims include diagnostic and procedural codes but are not a record of care received. There may be diagnoses which are preliminary when billed but later confirmed as an alternate diagnosis and patients may carry forward in time diagnoses from prior episodes of disease. We attempt to account for this by applying strict time criteria for defining episodes of disease. Second, because of the limited number of fields allowable per claim, these data do not provide comprehensive information of care received. For example, the MarketScan® claims data do not include facility or provider identifiers which would otherwise be important predictors of health care utilization. Nonetheless, claims do provide intricate structural and longitudinal patient-level data which may otherwise go unused in health services research. Third, prediction of future health care events in this study was structured around the concept of “low-value care” which we defined as surgery occurring after two or fewer episodes of diverticulitis. However, value of care may be perceived differently by patients. Determining how patients value the tradeoffs of elective surgery and symptom burden of diverticulitis is an active area of research in our group. Finally, prediction modeling should be interpreted with caution in the context of validation. Here we validated internally using repeated samples. Future iterations of these models should be validated using independent samples (i.e. external validation) [11].

Using each patient’s unique HCU data from around the time of diagnosis of diverticulitis, we trained machine learning models to predict future elective colon surgery, as far as months in advance. Of three models tested, the GBM demonstrated highest performance prediction which may be due to its ability to search out the most informative combinations of codes in HCU data. We also found that use of low-value surgery is driven by patients who are high utilizers of health care. By predicting future low-value surgery for a preference sensitive condition, we can inform shared decision making between patients and surgeons.

## Additional Files

The additional files for this article can be found as follows:

10.5334/egems.193.s1 Appendix A Cohort Selection.

10.5334/egems.193.s2 Appendix B Data Processing (Model Selection).

10.5334/egems.193.s3 Appendix C Data Preparation and Modeling Process.

10.5334/egems.193.s4 Appendix D Most influential codes for each model.
