# Supplementary material for: Predicting Future Elective Colon Resection for Diverticulitis Using Patterns of Health Care Utilization
Source: EGEMS (Wash DC). 2018 Jan 24;6(1):1. doi: 10.5334/egems.193 (PMC5983027; doi:10.5334/egems.193)
Supplement: Appendix A — Cohort Selection. [file egems-6-1-193-s1.pdf]

Appendix A: Cohort Selection

The following summarizes the steps applied in cohort selection and data processing prior to application of Machine Learning algorithms:

1. Using the entire MarketScan® database from 2009-2012, we selected all patients with a primary or non-primary diagnosis of diverticulitis (ICD-9: 562.11 or 562.13) on either inpatient or outpatient claims and identified the date for their first claim containing that diagnosis as their diagnosis date.
2. Using MarketScan® claims from 2007 onwards, patients were excluded if they did not have at least two years of continuous health insurance enrollment that was free from the diverticultis prior to their diagnosis date.
3. Patients who had any claims with procedural codes (CPT or ICD-9) indicating a colon surgery prior to the diagnosis of diverticulitis using the following codes:

Open Surgery Codes:

ICD-9

45.41	Excision of lesion or tissue of large intestine
45.7	Open and other partial excision of large intestine
45.71	Open and other multiple segmental resection of large intestine
45.72	Open and other cecectomy
45.73	Open and other right hemicolectomy
45.74	Open and other resection of transverse colon
45.75	Open and other left hemicolectomy
45.76	Open and other sigmoidectomy
45.79	Other and unspecified partial excision of large intestine
45.8	Total intra-abdominal colectomy
45.82	Open total intra-abdominal colectomy
45.83	Other and unspecified total intra-abdominal colectomy
45.92	Anastomosis of small intestine to rectal stump
45.93	Other small-to-large intestinal anastomosis
45.94	Large-to-large intestinal anastomosis
46.01	loop ileostomy
46.03	Exteriorization of large intestine
46.04	Resection of exteriorized segment of large intestine
46.1	Colostomy
46.10	Colostomy, not otherwise specified
46.11	Temporary colostomy
46.13	Permanent colostomy
46.14	Delayed opening of colostomy

46.2 Ileostomy
46.20 Ileostomy, not otherwise specified
46.21 Temporary ileostomy
46.22 Continent ileostomy
46.23 Other permanent ileostomy
46.24 Delayed opening of ileostomy
46.43 Other revision of stoma of large intestine
48.62 Anterior resection of rectum with synchronous colostomy
48.63 Other anterior resection of rectum

<u>CPT</u>
44110 excision of lesions of small or large intestine, single enterotomy
44111 excision of lesions of small or large intestine, multiple enterotomies
44130 Enteroenterostomy, anastomosis of intestine, with or without cutaneous enterostomy
44139 Mobilization of splenic flexure performed in conjunction with partial colectomy
44140 Colectomy, partial; with anastomosis
44141 Colectomy, partial; with skin level cecostomy or colostomy
44143 Colectomy, partial; with end colostomy and closure of distal segment (Hartmann)
44144 Colectomy, partial; with resection, with colostomy or ileostomy and creation of mucofistula
44145 Colectomy, partial; with coloproctostomy (low pelvic anastomosis)
44146 Colectomy, partial; with coloproctostomy (low pelvic anastomosis), with colostomy
44147 Colectomy, partial; abdominal and transanal approach
44150 Colectomy, total, abdominal, without proctectomy; with ileostomy or ileoproctostomy
44151 Colectomy, total, abdominal, without proctectomy; with continent ileostomy
44155 Colectomy, total, abdominal, with proctectomy; with ileostomy
44156 Colectomy, total, abdominal, with proctectomy; with continent ileostomy
44157 Colectomy, total, abdominal, with proctectomy; with ileoanal anastomosis, loop ileostomy
44158 Colectomy, total, abdominal, with proctectomy; with ileoanal anastomosis, creation of reservoir
44160 Colectomy, partial, with removal of terminal ileum with ileocolostomy
44320 Colostomy or skin level cecostomy

<u>Laparoscopic Surgery Codes</u>
<u>ICD-9</u>
17.3 Laparoscopic partial excision of large intestine
17.31 Laparoscopic multiple segmental resection of large intestine
17.32 Laparoscopic cecectomy
17.33 Laparoscopic right hemicolectomy
17.34 Laparoscopic resection of transverse colon

17.35 Laparoscopic left hemicolectomy
17.36 Laparoscopic sigmoidectomy
17.39 Other laparoscopic partial excision of large intestine
45.81 Laparoscopic total intra-abdominal colectomy
Or any ICD-9 Open code PLUS
54.21 Laparoscopy
54.51 Laparoscopic lysis of peritoneal adhesions

CPT
44187 Laparoscopy, surgical; ileostomy or jejunostomy, non-tube
44188 Laparoscopy, surgical, colostomy or skin level cecostomy
44204 Laparoscopy, surgical; colectomy, partial, with anastomosis
44205 Laparoscopy, surgical; colectomy, partial, with removal of terminal ileum with ileocolostomy
44206 Laparoscopy, surgical; colectomy, partial, with end colostomy (Hartmann)
44207 Laparoscopy, surgical; colectomy, partial, with end coloproctostomy
44208 Laparoscopy, surgical; colectomy, partial, with end coloproctostomy, with colostomy
44210 Lap, surgical; colectomy, total, abdominal, w/o proctectomy, with ileostomy or ileoproctostomy
44211 Laparoscopy, surgical; colectomy, total, abdominal, w/ proctectomy, with ileoanal anastomosis,
44212 Laparoscopy, surgical; colectomy, total, abdominal, with proctectomy, with ileostomy
44213 Laparoscopy, surgical, mobilization of splenic flexure performed with partial colectomy
44227 Lap, surgical, closure of enterostomy, large or small intestine, with resection and anastomosis
44238 Unlisted laparoscopy procedure, intestine

Or any CPT Open code PLUS
49320 Laparoscopy, abdomen peritoneum, omentum, diagnostic
44180 Laparoscopy, surgical enterolysis

4. Any patients who had a claim with a primary or non-primary diagnosis of colon cancer (ICD-9 153.9, 154.0, 154.1, 211.3) at any time during claim ascertainment (2007-2014) were excluded from the cohort.
5. Any patients who did not have at least two years of continuous health insurance enrollment after the date of diagnosis were excluded.
6. All primary components of health insurance claims were selected for inclusion in prediction models including diagnosis codes (ICD), procedural codes (CPT/HCPCS), drug codes (NDC) from claims fitting the time periods outlined in Figure 1.
