# Supplementary material for: Predicting Future Elective Colon Resection for Diverticulitis Using Patterns of Health Care Utilization
Source: EGEMS (Wash DC). 2018 Jan 24;6(1):1. doi: 10.5334/egems.193 (PMC5983027; doi:10.5334/egems.193)
Supplement: Appendix B — Data Processing (Model Selection). [file egems-6-1-193-s2.pdf]

Appendix B: Data Processing (Model Selection)

The following are links to source code for prediction models used in this study.

1. Random Forest: http://scikit-learn.org/stable/modules/generated/sklearn.ensemble.RandomForestClassifier.html https://github.com/scikit-learn/scikit-learn/blob/7f224f8f084019523982956786811dccb4092425/sklearn/ensemble/forest.py#L744
2. Penalized Logistic Regression: http://scikit-learn.org/stable/modules/generated/sklearn.linear_model.LogisticRegression.html https://github.com/scikit-learn/scikit-learn/blob/7f224f8f084019523982956786811dccb4092425/sklearn/linear_model/logistic.py#L1309
3. Gradient Boosting Machine: http://scikit-learn.org/stable/modules/generated/sklearn.ensemble.GradientBoostingClassifier.html https://github.com/scikit-learn/scikit-learn/blob/7f224f8f084019523982956786811dccb4092425/sklearn/ensemble/gradient_boosting.py#L1265
