# Supplementary material for: Predicting Future Elective Colon Resection for Diverticulitis Using Patterns of Health Care Utilization
Source: EGEMS (Wash DC). 2018 Jan 24;6(1):1. doi: 10.5334/egems.193 (PMC5983027; doi:10.5334/egems.193)
Supplement: Appendix C — Data Preparation and Modeling Process. [file egems-6-1-193-s3.pdf]

Appendix C: Data Preparation and Modeling Process

This supplement is presented in the form of a tutorial for reproducible research. However, the underlying Marketscan data files are proprietary and cannot be provided along with the data analysis codes.

Description of Data Preparation Process

The raw data from the Marketscan database is organized at the claim or encounter level. We begin our extract-transform-load process by working with each year of data separately, to reshape the claims data into complex sequence data for each individual. The full detail of this data processing is a tedious 249 lines of python code, listed here https://github.com/uwescience/debut/blob/master/etl.py, (to be made public on publication) and the key details are in the following loop:

```
for col in ['DX1', 'DX2', 'DX3', 'DX4', 'PROC1', 'NDCNUM']:
    dx = tt[col]
    if str(dx).strip() not in ['', 'nan']:
        # include prefix icd-, cpt-, and ndc- for
        # figuring out what is what later
        if col.startswith('DX'):
            dx = 'icd9-' + dx
        elif col == 'PROC1':
            dx = 'cpt-' + dx
        elif col == 'NDCNUM':
            dx = 'ndc-' + dx

        code_seq.append(dx)
```

The result of the etl.py script is a comma-separated-value file of “prepped_data” for a single year, with the following columns:

- id – unique patient id number
- year – data year
- age – patient age
- sex – 1 = male, 2 = female
- dvt – did diverticulitus code appear this year?
- emergency_dvt – was there any emergency care this year?
- dvt_surgery – was there a surgery for diverticulitis this year?
- fully_enrolled – was the patient enrolled in insurance for all months this year?
- code_seq – complex sequence of ICD, CPT, and NDC codes for all health care this year
- date_seq – date sequence corresponding to all codes in code_seq
- emrg_seq – 0/1 sequence indicating whether care was delivered in emergency setting

- out_seq – 0/1 sequence indicating whether care was outpatient
- in_seq – 0/1 sequence indicating whether care was inpatient

The etl.py script uses around 100 gigabytes of main memory and takes around 10 hours to run. It produces csv files of around 4 gigabytes for each year of data.

The next step in processing this data is to load all years (2007-2014) of processed csv data into memory and concatenate the yearly complex sequence data for each individual. This along with all “feature engineering” code is listed in the python code linked here https://github.com/uwescience/debut/blob/master/data.py, with the load function defined starting on line 21.

Description of Modeling Process

When the raw data from the Marketscan database has been transformed from complex sequence data into feature vectors, it is straightforward to use a number of machine learning methods from the scikit-learn python package to make predictions and evaluate (internal) out-of-sample validity with 10-fold hold-out cross-validation, and the code to do this is listed in https://github.com/uwescience/debut/blob/master/auc.py. Once the ETL process has been completed, the machine learning work takes around 50 lines of code, and can be listed entirely here:

```
import sys, numpy as np, pandas as pd
import sklearn.naive_bayes, sklearn.ensemble,
       sklearn.linear_model, sklearn.model_selection
import data, model

assert len(sys.argv) == 5, \
    'usage: python auc.py rep weeks ngram_min ngram_max'
rep = int(sys.argv[1])
weeks_after = int(sys.argv[2])
ngram_min = int(sys.argv[3])
ngram_max = int(sys.argv[4])
print(sys.argv)

# set random seed for reproducibility
np.random.seed(12345+rep)

# load data
patient_df, X, y = data.load_prepped_df(weeks_after,
                       ngram_range=(ngram_min, ngram_max))

# create dict of ML methods to consider
n_jobs = 10  # make sure to request resource level on cluster
clf_dict = {'GBM': sklearn.model_selection.GridSearchCV(
                       sklearn.ensemble.GradientBoostingClassifier(),
                       param_grid={
                           'max_depth': [1,3,5,7,9,11]},
```

```
                      n_jobs=n_jobs),
            'PLR': sklearn.linear_model.LogisticRegressionCV(
                       n_jobs=n_jobs, class_weight='balanced'),
            'RF': sklearn.ensemble.RandomForestClassifier(
                       n_estimators=100, n_jobs=n_jobs,
                       class_weight='balanced'),
           }

# run models and save results
all_results = pd.DataFrame()
for clf_name, clf in clf_dict.items():
    print(clf_name)
    sys.stdout.flush()

    results = model.auc_est(clf, X, y, [rep], range(10))
    results['clf_name'] = clf_name
    results['weeks_after'] = weeks_after
    print(results.auc.describe())
    all_results = all_results.append(results)

    # save results as you go, to fail faster
    dname = '/homes/abie/projects/2016/TICS/'
    all_results.to_csv(dname +
        'auc_results_{:02d}_{:02d}_{:d}-{:d}.csv'.format(
             rep, weeks_after, ngram_min, ngram_max),
        index=False)
```

Testing Process

There are many ways to make mistakes when doing complex ETL work like this (or even when fitting and evaluating models), and to provide some protection against errors from defective code, we implemented automatic tests of both data and model functions, following a “unit testing” pattern. The two final code listings linked in this supplement test the functions developed for loading data and fitting models:

- https://github.com/uwescience/debut/blob/master/test_model.py
- https://github.com/uwescience/debut/blob/master/test_data.py
