# Supplementary figures and images for: Predicting Future Elective Colon Resection for Diverticulitis Using Patterns of Health Care Utilization
Source: EGEMS (Wash DC). 2018 Jan 24;6(1):1. doi: 10.5334/egems.193 (PMC5983027; doi:10.5334/egems.193)

## Appendix D: Most influential codes for each model

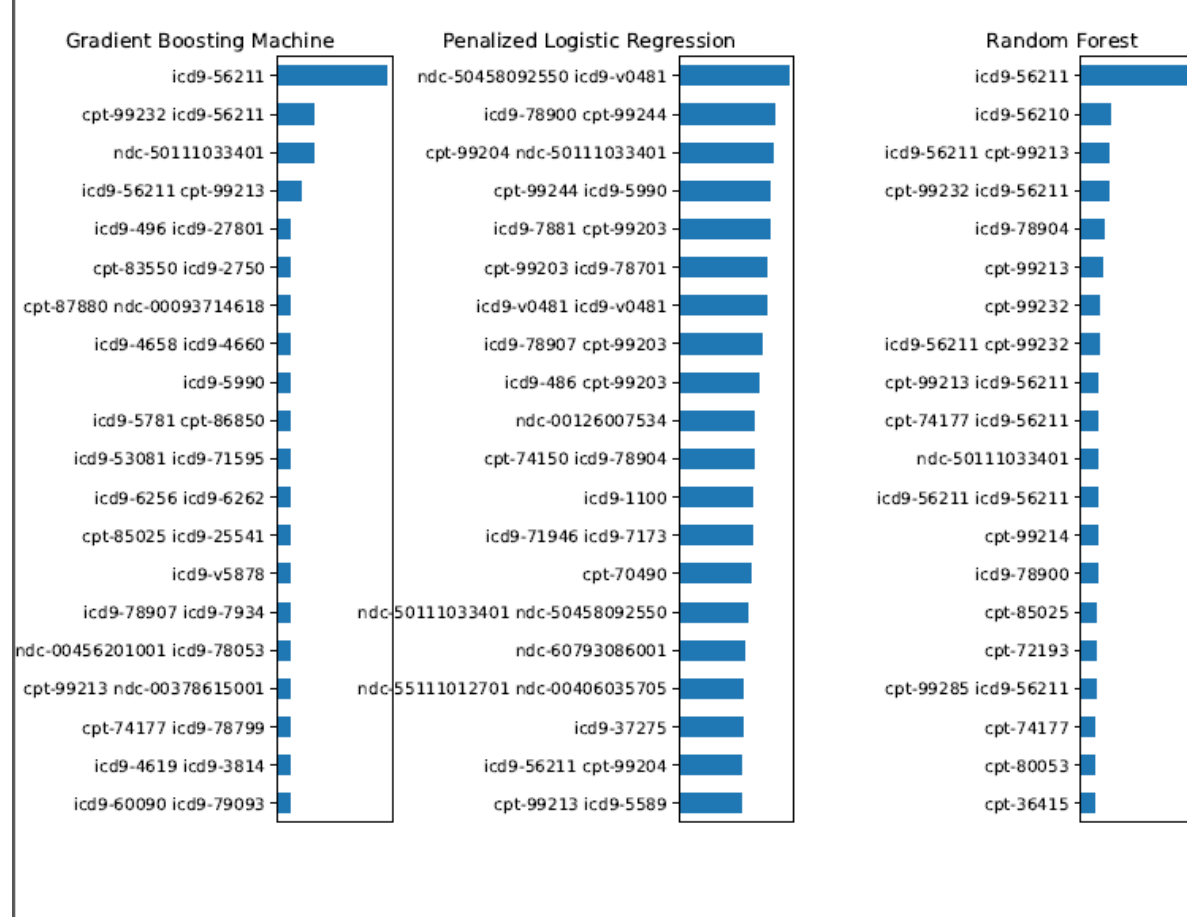

Supplement: Appendix D — Most influential codes for each model. [file egems-6-1-193-s4.pdf]
